# Calculation and program realization of coal pillar setting parameters in Huainan mining area

**DOI:** 10.1371/journal.pone.0297990

**Published:** 2024-02-29

**Authors:** Liangliang Yang

**Affiliations:** Surveying and Mapping Team, Huainan Jianfa Planning and Design Research Institute Co., Huainan, China; University 20 Aout 1955 skikda, Algeria, ALGERIA

## Abstract

Coal pillar retention plays a crucial role in ensuring safety and minimizing ground deformation in coal mining operations. However, accurately and efficiently determining the optimal size of protective pillars, reducing coal pillar pressure, and addressing challenges such as limited access to retention parameters, lengthy observation times, and high labor costs are challenges that must be addressed. In this paper, we presented a methodology using Huainan mine as a case study to address these challenges. The solution involves deriving the formula for coal pillar retention parameters based on the *Three Regulations* definition and requirements. The total least squares algorithm was integrated with surface observation station data and the MATLAB software platform to automate the coal pillar retention solution. Furthermore, a linear regression model of coal pillar retention-related parameters was established using the geological mining condition data. The proposed ELM neural network model was optimized using a genetic algorithm and combined with the linear regression model to establish a predictive model. The results demonstrated that the proposed machine learning algorithm attains the requisite level of accuracy for industrial production.

## Introduction

In the global landscape, energy stands as a cornerstone for economic development and societal progress. Among the various sources, coal continues to play a pivotal role, providing a substantial portion of the world’s energy needs. However, the challenges inherent in coal mining, such as unfinished pits, resource wastage, and potential risks to public safety, underscore the urgency of implementing effective and sustainable practices across energy-producing industries.

The prevailing "Regulations on Coal Pillars for Buildings, Water Bodies, Railways, and Major Shafts and Coal Pressure Mining" (commonly known as the "Three Regulations") have historically guided the reservation of protective coal pillars. Nevertheless, the lack of specificity within these regulations has resulted in suboptimal coal pressure on protective pillars, contributing to a significant loss of valuable coal resources.

Amidst these challenges, our research emerges as a beacon of scientific innovation, seeking to address critical gaps in current coal mining practices. This study stands at the forefront of scientific exploration, offering novel insights and methodologies to revolutionize the approach towards coal pillar design and protective measures. As we navigate the complexities of energy production, our work pioneers a path towards more efficient, safe, and sustainable coal mining operations.

Recognizing the global significance of energy and the broader implications of inefficient coal mining practices, recent advancements have led to the introduction of new regulations to replace the outdated "Three Regulations." These updated guidelines mandate mining areas worldwide to define specific parameters for protective coal pillars based on unique geological conditions. By doing so, these regulations aim to harmonize safe production, environmental preservation, and resource conservation in the diverse contexts of energy-producing regions.

This study, focused on the Huainan coal mining region, seeks to calculate and implement parameters for setting protective coal pillars. Building upon the pioneering work of researchers in this field, such as Zhang [1], who developed functions related to coal pillar retention using the ArcEngine software platform and the C# programming language, and Wang et al. [2], who established a three-dimensional numerical model using actual geological data and the Mohr–Coulomb model, our research employs theoretical analysis, FLAC3D numerical simulations, and stress monitoring, as demonstrated by Meng et al. [3]. These collective studies have provided practical support for retaining coal pillars in deep wells with thick coal seams, meeting safety mining requirements, and minimizing the wastage of coal resources [1–5].

Moreover, the findings of this research are expected to resonate across various energy-producing industries, transcending geographical constraints. Our work contributes valuable insights that can inform policy decisions and industry practices not only in the Huainan region but globally. Ultimately, this research plays a pivotal role in advancing the efficiency, safety, and sustainability of energy-producing operations, addressing key challenges that extend beyond borders and supporting the long-term development goals of nations worldwide.

Apart from its economic significance, the calculation of coal pillar retention is crucial for ensuring safety during the coal production process. However, determining the relevant parameters can be challenging due to considerable variations in coal pillar retention parameters across different geological formations. In addition, setting up surface observation stations must be set up in different areas affected by surface mining, and coal pillar retention parameters must be determined through prolonged observation and calculation. This results in a lengthy operation time and high economic costs. With the advancement of machine learning (ML) algorithms, various methods are now available for inferring challenging observation data by using readily available data, which greatly enhances work efficiency. He et al. [6] analyzed the geological formation of thick alluvial layers through theoretical analysis. They collected surface observatory data and used them to establish a genetic algorithm (GA) for probabilistic integral parameter inversion, solving the problem of probabilistic integral parameter inversion in multi-coal seam and multi-working face mining, thereby providing a quicker and more accurate determination of the surface movement deformation law. Ma [7] examined the hierarchical structure of coal processing equipment, focusing on monitoring the jigging process; a support vector machine (SVM) model optimized using a GA was constructed, demonstrating the superior accuracy of ML algorithms in handling complex data based on user experience and diagnostic results. In a previous study [8], a hybrid strategy of using a GA model and a BP neural network model was established through data analysis and research on the relationship of the comprehensive mining working face system. This approach enables optimal process parameter selection and assists mine leadership in decision analysis. These studies have practical implications [6–9] as they provide valuable insights into mining operations and can aid in decision-making in the mining industry.

This article also conducts an in-depth analysis of coal seam gas permeability, which is a coupling process between gas migration and coal seam deformation. The permeability of coal seams is not only related to factors such as the structural characteristics of the coal itself, but is also affected by the environmental factors of the geophysical field (geo-stress field, geo-temperature field, gas field, etc.) in which it is located. Coal gas permeability is a parameter that reflects the ease of gas flow in the coal seam, and its measurement plays an important role in solving mine safety problems. The change of coal gas permeability is an important issue. In order to study the changes in coal permeability, some scholars have conducted a series of studies and achieved many results. Lin and Zhou [10] studied the permeability of coal sample gas by simulating the in-situ stress environment, and obtained the relationship between coal gas permeability and in-situ stress. Zhao et al. [11] conducted a coal sample permeability test under three-dimensional stress and revealed the influence of three-dimensional stress and coal adsorption on the coal gas seepage law. Tang et al. [12] obtained the relationship between permeability and effective stress by studying the desorption and migration laws of coal seam gas during the simulated gas drainage process. Research by Enever and Hennig [13] pointed out that the change in coal permeability has an exponential relationship with the change in in-situ stress. Yin et al. [14] and Jiang et al. [15] used the self-developed "gas-containing coal thermal fluid-solid coupling three-axis servo seepage experimental device" to conduct a large number of gas seepage experiments under different conditions, and obtained the relationship between permeability, gas pressure, and ground stress. relation. Xu et al. [16, 17] took briquettes and raw coal as research objects respectively, and discussed the influence of temperature on the mechanical properties and seepage characteristics of coal samples under triaxial stress conditions. Liang et al. [18, 19] took rocks as the research object and studied the relationship between temperature and permeability. Previous research has played a very useful role in promoting the solution of this problem. Despite this, due to the multi-factor, complexity, randomness and fuzziness of coal gas permeability, its more accurate prediction method still needs to be further explored and revealed. In recent years, some scholars have begun to use artificial neural networks to solve mine safety problems that are highly nonlinear, complex and ambiguous [14–19]. Artificial neural network is an intelligent information processing system that imitates the structure and function of the human brain. It has strong adaptive learning capabilities, parallel information processing capabilities, fault tolerance and nonlinear function approximation capabilities. It is used to solve problems with multi-factor problems, complexity, randomness and nonlinear problems provide a new approach. Error back propagation neural network (error back propagation neural network), also known as BP algorithm [20], is one of the most important networks in neural networks. It has strong nonlinear dynamic processing capabilities and can be implemented without knowing the relationship between input and output. A highly nonlinear mapping [21], suitable for extracting features from sample data, and better expressing the implicit nonlinear correspondence between each input and output [22]. However, it should be noted that the performance of the model also depends on the quality [23], size and characteristics of the data, as well as the correct configuration and parameter tuning of the algorithm [24]. By analyzing the relationship between various influencing factors and coal mass permeability, and based on a large amount of test data obtained in the laboratory, a BP neural network was used to establish a coal gas permeability prediction model that takes into account the structural characteristics of the coal mass itself and environmental factors. Traditional models are often limited by linear assumptions and feature engineering challenges, making it difficult to achieve the same performance level.

Recent advancements in the field have sought to address these challenges. Vu and Do (2023) conducted a study titled "Determination of the rock mass displacement zone by numerical modeling method when exploiting the longwall at the Nui Beo Coal Mine, Vietnam." This research, published in the Mining of Mineral Deposits journal, explores the numerical modeling method to determine the rock mass displacement zone during longwall exploitation, providing valuable insights into the efficient and safe extraction of coal resources [25].

Furthermore, Bazaluk et al. (2023) investigated the "Impact of ground surface subsidence caused by underground coal mining on natural gas pipeline." Published in Scientific Reports, their study delves into the consequences of ground surface subsidence resulting from underground coal mining on natural gas pipelines. This research sheds light on the broader implications of mining activities on infrastructure and environmental factors [26].

In contrast, the hybrid neural network prediction model proposed in this article makes full use of the nonlinear modeling capabilities of neural networks and combines global optimization and adaptive techniques to perform in a more accurate, robust and adaptable manner. Coal seam permeability prediction.

In this paper, we focused on the solution of coal pillar retention parameters. We presented two types of parameter solution methods based on the geological structure conditions of thick loose layers in the Huainan mining area. The first method involves combining the “Three Regulations” to construct an equation that relates coal pillar retention parameters when the data from surface observation stations are highly accurate. The second method involves establishing an extreme learning machine (ELM) model optimized using a GA and mathematical regression to predict the relevant parameters through ML when highly accurate geological mining conditions data are available.

## Methods

### Determination of coal pillar retention parameters

To determine the coal pillar retention parameters, two observation routes are deployed on the surface according to the mining direction and range of the underground working face. Corresponding surface mobile observation stations are set up to collect observation data, and analogous reasoning is used to determine the parameters. However, in some locations, the deployment of observation stations for data collection can be challenging and time-consuming. The observatory adopts a profile-shaped observation station. Set in a straight line and perpendicular or parallel to the coal seam trend, each observation line should be set with at least one control point. The designed observatory should be calibrated to the field by: design the observatory on the (mining engineering plane) (upper and lower comparison map) map, in order to use GPS to determine the temporary control point from the control point of the mining area near the observation station, through the control point to calibrate each observation point in turn, and give a number, the requirements for the buried point are: during the observation period can be reliably stored, and firmly combined with the surface for easy observation, generally higher than the ground 10 ~ 20cm, in higher areas should consider the measurement point after sinking after the possible reserved part should be higher.

When burying the point, dig a pit with a diameter of 0.2~0.3m and a depth of not less than 0.6m in the calibrated position, pour it with concrete, use a 1620mm iron rod as a mark in the middle, and process the top of the middle into a spherical engraved cross trough as the center of the measurement point mark. The watering depth of the mark reference point should be about 0.5m below the topsoil layer, and the surrounding area should be filled with earth and rock to prevent other impacts on the measurement point. To ensure accurate data, the basic content of the observation work of the surface mobile observation station is to periodically and repeatedly measure the changes in the spatial position of each measurement point on the observation line in different periods during the acquisition process. Specifically, it can be divided into connection measurement of observation stations, comprehensive observation, level measurement carried out separately, measurement and cataloging of surface damage.

Therefore, in this paper, we derived a solution formula [27–30] for coal pillar retention parameters based on the conditions and guidelines included in the “Three Regulations”.

Consider the integrated boundary angle as an example. The bedrock boundary angle is determined by projecting the boundary point with a sinking of 10 mm along the loose layer boundary angle onto the bedrock surface. The angle between the projected point and the boundary of the mining area and the horizontal plane on the side of the coal pillar is calculated. For the integrated movement angle, the outermost boundary point is projected along the loose layer movement angle onto the bedrock surface. The angle between the line of the projection point and the boundary of the mining area and the horizontal plane on the side of the coal pillar is the bedrock movement angle. The parameters used in this calculation are as follows: *i* = 3 mm/m, *K* = 0.2 mm/m^2^, and *ε* = 2 mm/m.

**Fig 1:**
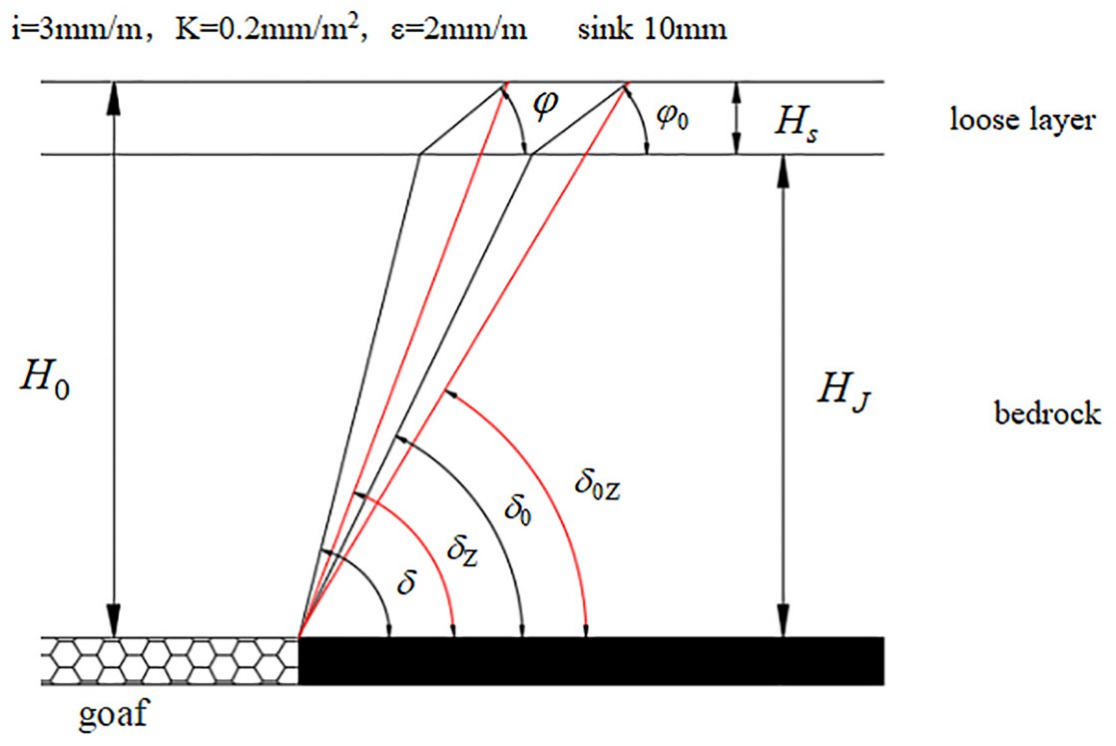
Determination of the angular parameter of the loose layer and bedrock (direction).

**Fig 2:**
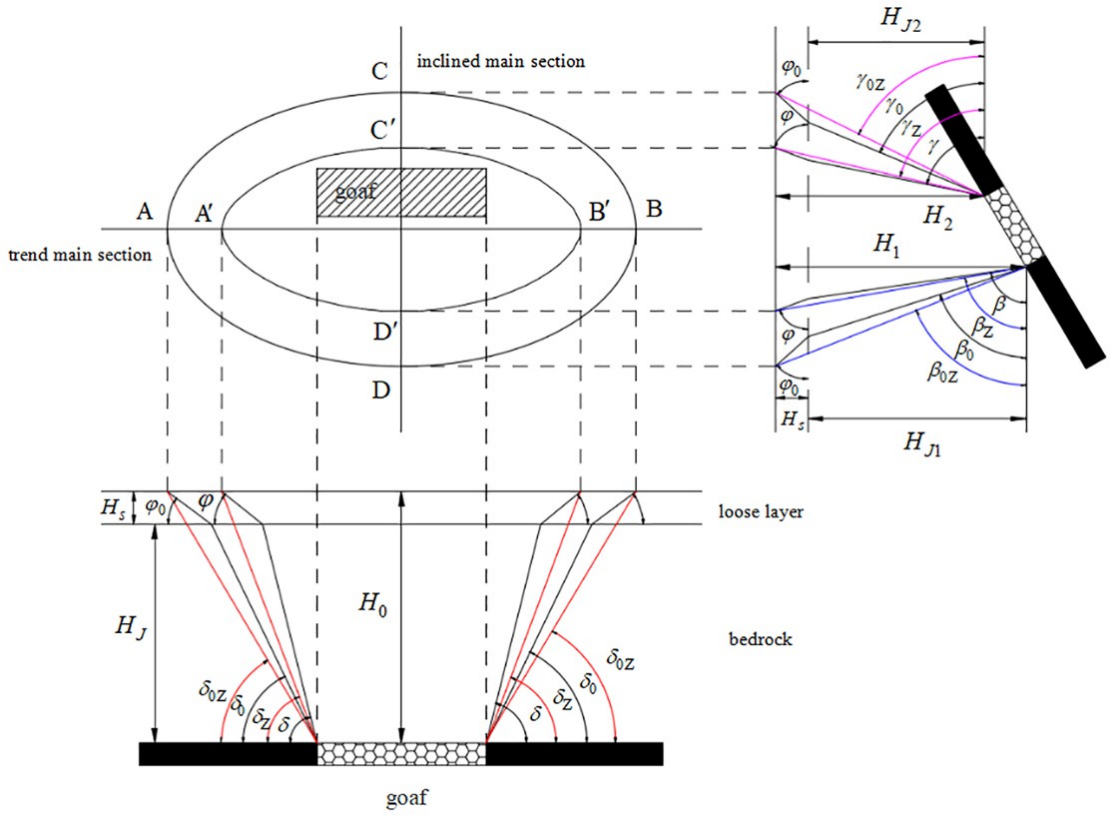
Relationship of coal pillar retention parameters. (Note: In Fig 2, *φ*_0_ and *φ* are the loose layer boundary angle and movement angle, respectively; H_*S*_, H_*J*_, H_*J*1_, and H_*J*2_ are the loose layer thickness, bedrock thickness, lower mountain bedrock thickness, and upper mountain bedrock thickness, respectively; H_0_, H_1_, and H_2_ are the average mining depth, lower mountain average mining depth, and upper mountain average mining depth, respectively; *β*_0Z_, *γ*_0z_, and δ_0Z_ are the integrated lower mountain boundary angle, integrated upper mountain boundary angle, and integrated strike boundary angle, respectively; *β*_*Z*_, *γ*_*Z*_, and *δ*_*Z*_ are the integrated lower mountain movement angle, integrated upper mountain movement angle, and integrated strike movement angle, respectively; *β*_0_, *γ*_0_, and *δ*_0_ are the integrated lower mountain bedrock boundary angle, integrated upper mountain bedrock boundary angle, and integrated strike bedrock boundary angle, respectively; are the integrated lower mountain bedrock boundary angle, integrated upper mountain bedrock boundary angle, and integrated strike bedrock boundary angle boundary angle, respectively; *β*, *γ*, and *δ* are the lower mountain bedrock movement angle, upper mountain bedrock movement angle, and strike bedrock movement angle, respectively).

From the comparison shown in Fig 1, we can derive the calculation formulas for the strike boundary angle and the movement angle according to the definition of the integrated boundary angle and the analytical description provided in Fig 2:

$$H_{0}\;\text{cot}\;\delta_{0Z}=H_{S}\;\text{cot}\;\varphi_{0}+H_{J}\;\text{cot}\;\delta_{0},$$ (1)

$$H_{0}\;\text{cot}\;\delta_{Z}=H_{S}\;\text{cot}\;\varphi +H_{J}\;\text{cot}\;\delta .$$ (2)

Similarly, by using Eqs (1) and (2), and as shown in Fig 2, we can derive the following formulas:

The calculation formulas for the downhill boundary angle and the moving angle:

$$H_{1}\;\text{cot}\;\beta_{0Z}=H_{S}\;\text{cot}\;\varphi_{0}+H_{\text{J}1}\;\text{cot}\;\beta_{0},$$ (3)

$$H_{1}\;\text{cot}\;\beta_{Z}=H_{S}\;\text{cot}\;\varphi +H_{\text{J}1}\;\text{cot}\;\beta .$$ (4)

The calculation formulas for the uphill boundary angle and the moving angle:

$$H_{2}\;\text{cot}\;\gamma_{0Z}=H_{S}\;\text{cot}\;\varphi_{0}+H_{\text{J}2}\;\text{cot}\;\gamma_{0},$$ (5)

$$H_{2}\;\text{cot}\;\gamma_{Z}=H_{S}\;\text{cot}\;\varphi +H_{\text{J}2}\;\text{cot}\;\gamma .$$ (6)

The parameters in Eqs (1)–(6) are defined as previously mentioned. By using these six formulas, the unknown parameters of the coal pillar can be determined using geological data. However, the calculation process involved in determining these parameters is complex and requires extensive data processing; this can result in the introduction of errors and excessive consumption of valuable time and resources. The calculation formulas for deriving strike boundary angles, moving angles, downhill boundary angles, and uphill boundary angles from geological data provide us with a powerful tool for better understanding subsurface geological structures. These formulas allow us to accurately calculate these key angles by looking at information such as magnetic azimuth, inclination, etc. in geological data, which is helpful in the study of coal columns or other stratigraphic features and resource exploration. Accurate measurements of these angles are essential for further research and development in the fields of geology and geoengineering.

To address this problem, in this study, we used the commonly used least squares method and the MATLAB software platform to implement the necessary programming for the protective coal pillar retention parameters.

### Programming implementation

To minimize the correction error in the mathematical model and eliminate the problem arising from matrix errors, we implemented the parameter solving process by using the total least squares (TLS) algorithm in the MATLAB platform to realize the automation of the coal pillar retention parameter solving process.

#### Total least squares algorithm

**Fig 3:**
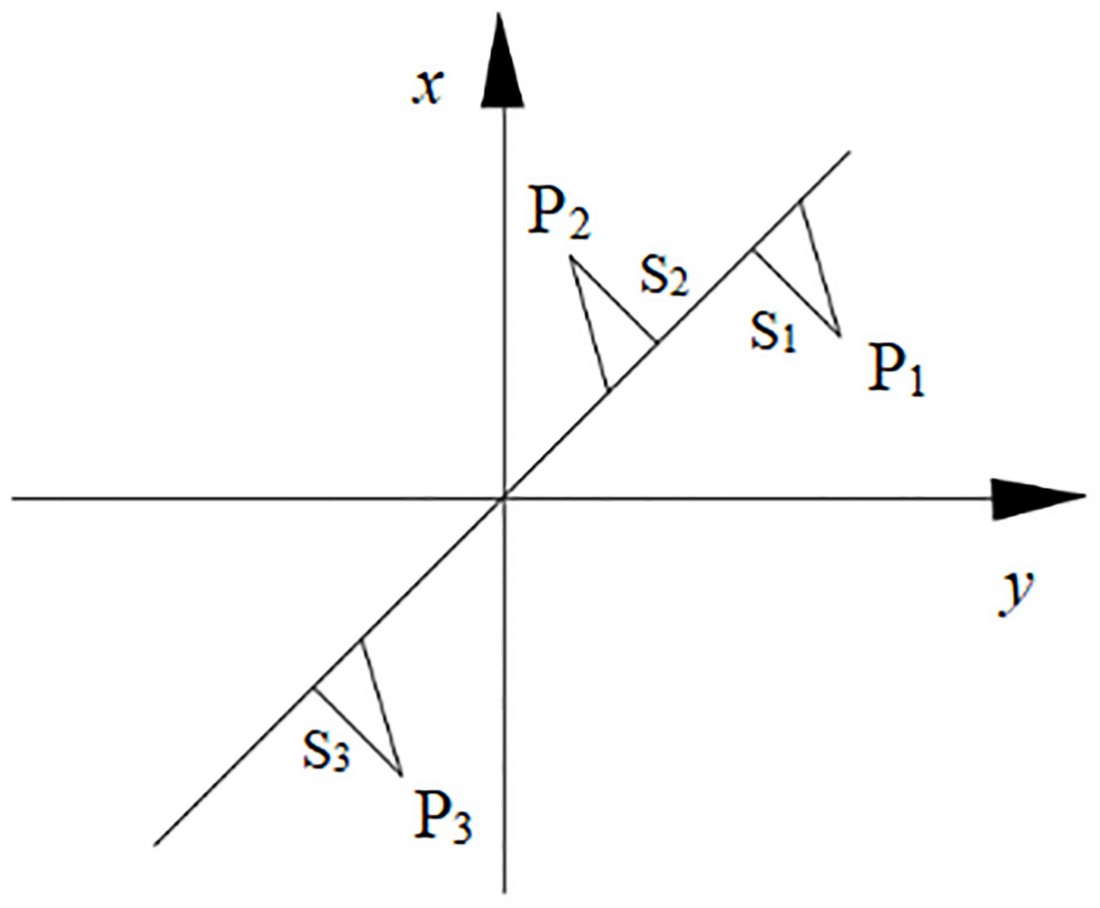
Geometric significance.


*Basic principle*. The TLS algorithm was proposed by Golub and VanLoan in 1980; they obtained the TLS solution for the error-in-variable (EIV) model by using the singular value decomposition (SVD) method by performing a numerical analysis and provided a detailed description and analysis of this novel leveling method. The geometric significance of the TLS leveling criterion is illustrated in Fig 3 [31–33].

$$L=A\hat{X}$$ (7)

In Eq (7), *L* represents the observation vector of *n*×*l*, and *A* represents the *n*×*m* coefficient matrix, with *rank(A)* = *n < m*.

Simultaneously considering the errors in the coefficient matrix and the observations, the EIV model is used to represent the functional model as follows:

$$\left(A+E_{A}\right)X=L+e_{L},$$ (8)

Where *E*_*A*_ is the error matrix of the coefficient matrix A, and *e*_*L*_ is the error vector of the observation vector L.

The mean and covariance array of error vectors is

$$\left[\begin{array}{c} e_{L}\\ e_{A} \end{array}\right]\sim N\left(\begin{array}{cc} \begin{array}{c} 0\\ 0 \end{array} & \sigma_{0}^{2}\left[\begin{array}{cc} Q_{L} & 0\\ 0 & Q_{A} \end{array}\right] \end{array}\right),$$ (9)

Where *e*_*A*_ = *vec*(*E*_*A*_), where *vec* represents the straightening operation of the matrix, *Q*_*L*_ refers to the covariance array of *e*_*L*_ (i.e., *mn×mn* order), *σ*_0_ represents the error in unit power, and $Q_{L}=P_{L}^{-1}$, $Q_{A}=P_{A}^{-1}$.

If the accuracy of the coefficient matrix and the observation vector are equal, then

$$Q_{L}=I_{m},Q_{A}=I_{mn},$$ (10)

where *I*_*m*_ represents the unit matrix of order m, *I*_*m*_ represents the unit matrix of order mn, and *I*_*m*_ = *I*_*m*_ ⊗*I*_*n*_, where ⊗ represents the Kronecker product (Kronecker–Zeh-fuss product).

Because the EIV model considers the errors in the coefficient matrix A and observation vector L, the TLS algorithm can be used to obtain the mathematical function of the EIV model:

$$e_{L}^{T}Q_{L}^{-1}e_{L}+e_{A}^{T}Q_{A}^{-1}=min.$$ (11)

By using Eq (11), the optimal expected values of *e*_*L*_, *e*_*A*_, and *X* can be obtained. If *Q*_*L*_ and *Q*_*A*_ satisfy Eq (10), it means that this is an equal-weighted TLS; as such, the weights can be disregarded.

*Solution method*. There are two main methods for solving the TLS problem: the SVD method and the Euler–Lagrange approximation iteration method. In this paper, we used the SVD algorithm to program the solution.

Transforming Eq (7), we obtain:

$$\left[\begin{array}{cc} \text{A} & \text{L} \end{array}\right]\left[\begin{array}{c} \text{X}\\ -1 \end{array}\right]=0,$$ (12)

where

A ∈ R^m×n^, L ∈ R^m^, x ∈ R^n^, and m > n, rank(A) = n.

Let the augmented matrix be $C=\left[\begin{array}{cc} A & L \end{array}\right]$. the augmented matrix obtained by solving is $\hat{C}=\left[\begin{array}{cc} \hat{A} & \hat{L} \end{array}\right]$, and then perform singular value decomposition on the augmented matrix, yielding: $\text{C}=\text{U}\sum {\text{V}}^{\text{T}},$ where Σ = *diag*(*σ*_1_, *σ*_2_, ⋯ *σ*_*n*_, *σ*_*n*+1_) and *σ*_1_ ≥ *σ*_2_ ≥ ⋯ ≥ *σ*_*n*_ ≥ *σ*_*n*+1_.

Because *σ*_*n*+1_ ≠ 0, the rank of the augmented matrix *C* can be expressed as (*n* + 1). In this case, the equation $\left[\begin{array}{cc} A & L \end{array}\right]{\left[\begin{array}{cc} x^{T} & -1 \end{array}\right]}^{T}\approx 0$ is a contradiction equation. To obtain the TLS result, the rank of the augmentation matrix $\hat{C}$ should be *n*, not (*n* + 1). According to the Eckart–Young–Mirsky principle, the best approximation matrix $\left[\begin{array}{cc} \hat{A} & \hat{L} \end{array}\right]$ of $\left[\begin{array}{cc} A & L \end{array}\right]$ should satisfy the following condition:

$$\left[\begin{array}{cc} \hat{\text{A}} & \hat{\text{L}} \end{array}\right]=\text{U}\sum {\hat{\text{U}}}^{\text{T}},$$

Where Σ = diag(*σ*_1_, *σ*_2_, ⋯ *σ*_*n*_, *0*), $\left[\begin{array}{cc} \hat{A} & \hat{L} \end{array}\right]=\hat{U}\sum {\hat{U}}^{T},\sum =diag({\hat{\sigma }}_{1},{\hat{\sigma }}_{2},\cdots {\hat{\sigma }}_{n},0)$.

The TLS corrections must satisfy the following conditions:

$$\sigma_{n+1}=\begin{array}{c} \text{min}\\ rank\left(\left[\begin{array}{cc} \hat{A} & \hat{L} \end{array}\right]\right) \end{array}{\left\|\left[\begin{array}{cc} A & L \end{array}\right]-\left[\begin{array}{cc} \hat{A} & \hat{L} \end{array}\right]\right\|}_{F},$$

$$\left[\begin{array}{cc} A & L \end{array}\right]-\left[\begin{array}{cc} \hat{A} & \hat{L} \end{array}\right]=\left[\begin{array}{cc} E_{A} & E_{L} \end{array}\right]=\sigma_{n+1}u_{n+1}v_{n+1}^{T},$$ (13)

Where *u*_*n*+1_ represents the (*n* + 1)th column of the orthogonal matrix *U*, and *v*_*n*+1_ represents the (*n* + 1)th column of the orthogonal matrix *V*.

The correction $\left[\begin{array}{cc} E_{A} & E_{L} \end{array}\right]$ in Eq (13) has rank 1, thus yielding $\left[\begin{array}{cc} A & L \end{array}\right]{\left[\begin{array}{cc} x^{T} & -1 \end{array}\right]}^{T}=0$.

From the above solution, it is evident that the last column of the singular vector *v*_*n*+1_ on the right side of the augmented matrix is the solution of the TLS: ${\left[\begin{array}{cc} {\hat{x}}^{T} & -1 \end{array}\right]}^{T}=\frac{-1}{v_{n+1,n+1}}v_{n+1}$.

Upon classification, we obtain $\hat{\text{x}}=-\frac{1}{{\text{v}}_{\text{n}+1,\text{n}+1}}\left[{\text{v}}_{1,\text{n}+1},{\text{v}}_{2,\text{n}+1},\cdots {\text{v}}_{\text{n},\text{n}+1}\right]$.

#### Implementation of parameter solving procedure

Data from a single observatory yields six equations, and according to the equation for resolving the coal pillar retention parameters, a minimum of two observatories is required to solve for eight unknowns. In this paper, we developed an algorithm [34, 35] for the integrated solution of multiple observation stations by utilizing the TLS criterion to increase the precision of parameter solutions and expand the scope of mathematical model development.

Case study:

Within the Huainan mine, there is a protected building. The geological characteristics of the mine consist of thick loose seams, with coal seam inclination angles of 2°–15°. The mine employs the collapse method as the roof management method. Data from six observation stations were collected (Table 1).

**Table 1:** Observation station data.

	1#	2#	3#	4#	5#	6#
***H***_***S***_	455	524	470	450	400	439.7
***H***_***J***_	435	150	120	125.4	371.5	88.3
***H***_**J1**_	435	150	160	125.4	420	88.3
***H***_**J2**_	435	150	80	125.4	323	88.3
***H***_**0**_	890	674	590	575.4	771.5	528
***H***_**1**_	890	674	630	575.4	820	528
***H***_**2**_	890	674	550	575.4	723	528
*δ*_0Z_	54.3	50.6	42.7	42.7	57	46.2
*β*_0Z_	49.3	49.4	48.1	48	48.9	42.8
γ_0Z_	51	49.7	48.1	48	46.7	42.8
δ_Z_	70.7	67.9	60.9	60.9	77.7	78.4
β_*Z*_	67.3	66.4	65.6	65.6	77.1	66.6
*γ*_Z_	69.6	62.9	65.6	65.6	75.3	66.6

**Fig 4:**
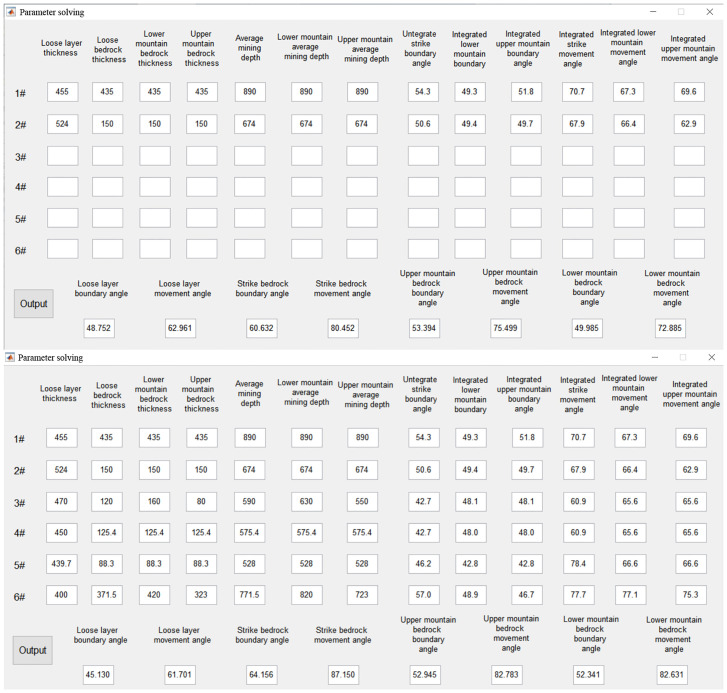
Parameter solving.

In this paper, two sets of data and equations were used to obtain the coal pillar retention parameters. The results are shown in Fig 4.

To validate the accuracy of the TLS algorithm, the conventional least squares algorithm was used in the calculation process to obtain the solution, and the accuracy of the results was compared using the median error value (The results and the medium error data are seen in Table 2).

**Table 2:** Results and medium error.

Solution Method	Integral least squares algorithm				Least squares algorithm			
	2 sets of data		6 sets of data		2 sets of data		6 sets of data	
	Results	Medium Error	Results	Medium Error	Results	Medium Error	Results	Medium error
*φ*_0_	62.9605	0.0107	45.132	0.0213	62.9208	0.3454	44.8232	0.563
*φ*	48.7516	0.0107	61.7055	0.0213	48.6306	0.3454	61.3644	0.563
*δ*_0_	60.6324	0.0198	64.1558	0.0492	60.9273	0.7927	61.7813	1.3818
*δ*	80.4523	0.0198	87.1501	0.0492	80.5470	0.7927	79.7804	1.3818
*γ*_0_	53.3937	0.0198	52.9447	0.0518	53.6662	0.7927	59.7717	1.3853
*γ*	75.4988	0.0198	82.7828	0.0518	75.6063	0.7927	78.8542	1.3853
*β*_0_	49.9850	0.0198	52.3409	0.0464	50.2440	0.7927	55.9917	1.2285
*β*	72.8864	0.0198	82.6309	0.0464	72.9978	0.7927	76.2948	1.2285

As can be observed from the data presented in Table 2, the TLS algorithm exhibited lower error values compared to the commonly used least squares algorithm. However, as the number of observatories involved in the calculation increased, there was a noticeable trend toward higher error values. The geological data collected by each observatory varied considerably with an increase in the range of the mining area. This decrease in the accuracy of the parameter solution highlights the importance of considering the geological conditions of a smaller area as a whole, rather than a larger area. Therefore, to obtain optimal results, the range of the measurement area must be controlled, and mining areas with similar geological conditions and located at close distances must be used.

### Construction of the proposed prediction model

By collecting the measurement data from the surface observatory of the working face in the Huainan mining area, a linear fitting analysis of the coal pillar retention parameters was conducted using the geological data, and a prediction model for coal pillar retention parameters was constructed using a GA and ELM [5, 36–38].

#### Regression model

A regression mathematical model was developed to determine the relationship between the coal pillar retention parameters, namely the downhill movement angle, uphill movement angle, strike movement angle, downhill boundary angle, uphill boundary angle, and strike boundary angle, and geological factors, namely the thickness of loose seam, coal mining depth, coal seam thickness, and bedrock thickness, by performing a regression analysis based on the collected measurement data. The established mathematical model was analyzed to determine the underlying laws, thereby providing a framework for the construction of a prediction model for coal pillar retention parameters in future research.

**Fig 5:**
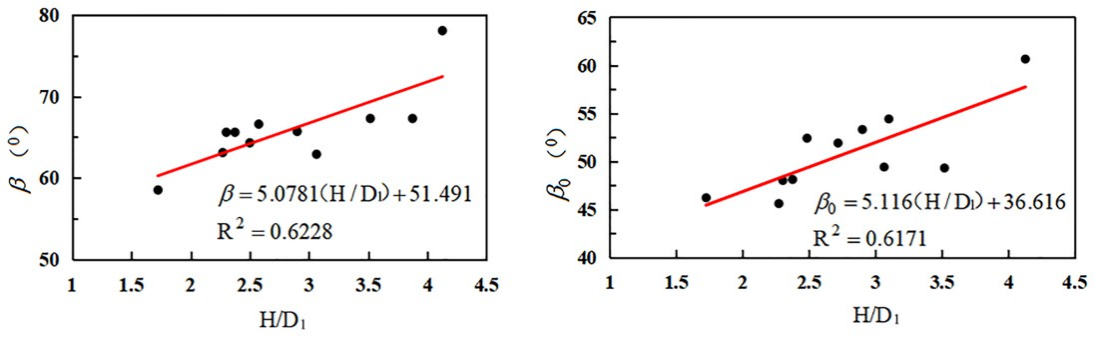
Relationship between downhill movement angle, boundary angle, and mining depth/working face width.

(1) The downhill movement angle and boundary angle were found to be primarily associated with the mining depth and width of the working face (Fig 5 and Table 3).

**Table 3:** Downhill movement angle, boundary angle, and related parameters.

Observation station	*H*(m)	*D*_1_(m)	*β*(°)	*β*_0_°)
***1242(1)	781	252	-	54.4
***1111(3)	570	240	65.6	48.1
***1311(1)	833	215	67.3	49.3
***12326	557	205	68.6	51.88
***12126	524	211	58.25	52.38
***1222(1)	945	229	78.13	60.63
***1242(3)	626	363	58.5	46.2
***11118	498.8	172	65.7	53.3
***11418	530	212	64.3	45
***1262(1)	890	253	67.3	49.3
***1141(3)	674	220	62.9	49.4
***1312(1)	528	205	66.6	42.8
***1232(3)	575.4	250	65.6	48
***11125	391	264	66.9	53.8
***1111(3)	477	210	63.1	45.6

As can be seen from Fig 5, the downhill movement angle and downhill boundary angle exhibited an upward trend with the increase in the ratio of mining depth to working face width.

(2) The uphill movement angle and upper hill boundary angle were found to be mainly related to the mining depth of the working face and the thickness of the loose layer of the working face (Table 4).

**Table 4:** Moving angle and boundary angle of the upper hill and related parameters.

Observation station	*H*(m)	*h*	*γ*(°)	*γ*_0_(°)
***1242(1)	781	165	84.9	-
***1111(3)	570	470	65.6	48.1
***1111(1)	920	278	69.5	47
***1311(1)	833	530	69.6	51.8
***12326	557	448	63	41.93
***1222(1)	945	321	78.13	60.63
***1611(3)	473	385	-	43.58
***1117(1)	771.5	400	75.3	46.7
***11418	530	387.5	68.6	49
***1262(1)	890	455	69.6	51.8
***1141(3)	674	524	66.4	49.7
***1312(1)	528	439.7	66.6	42.8
***1232(3)	575.4	450	65.6	48
***11125	391	156	66.9	53.8
***1111(3)	477	311	65.5	49.4

**Fig 6:**
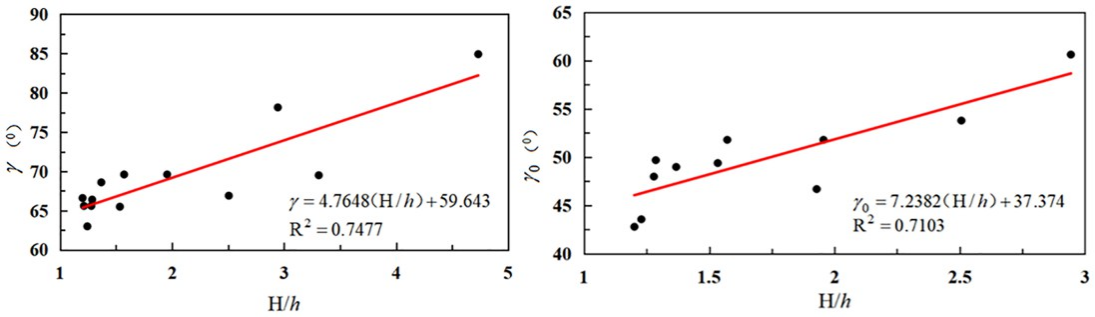
Relationship between uphill movement angle, boundary angle, and mining depth/thickness of the loose layer.

As can be seen from Fig 6, when the ratio of working face width to loose layer thickness increased, the uphill moving angle and uphill boundary angle increased.

(3) The mining depth of the working face, breadth of the working face, and thickness of the loose layer were found to be primarily influenced by the strike boundary angle and movement angle. The associated data and mathematical regression model are presented in Table 5.

**Table 5:** Uphill mountain movement angle, boundary angle, and related parameters.

Observation station	*H*(m)	*D*_1_	*h*	*δ*(°)	*δ*_0_(°)
***1242(1)	781	252	165	86.9	58
***1252(1)	802	269	165	81	54.9
***1111(3)	570	240	470	60.9	42.7
***1111(1)	920	220	278	79.9	58.1
***1311(1)	833	215	530	70.7	54.3
***12326	557	205	448	56.9	41.75
***12126	524	211	450	59	44.42
***1222(1)	945	229	321	74.5	56.4
***1242(3)	626	363	336	66.4	46.1
***1611(3)	473	-	385	-	46.7
***11118	498.8	172	403	70.7	55.3
***1117(1)	771.5	240	400	77.7	57
***11418	530	212	387.5	67.6	58
***1414(1)	725	251	415	55.4	41.8
***1262(1)	890	253	455	70.7	54.3
***1141(3)	674	220	524	67.9	50.6
***1312(1)	528	205	439.7	78.4	46.2
***1232(3)	575.4	250	450	60.9	42.7
***11125	391	264	156	66.9	53.8
***1111(3)	477	210	311	64.5	48.3

**Fig 7:**
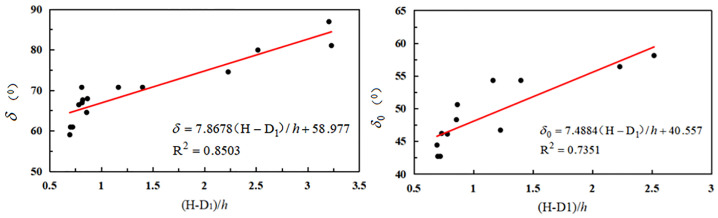
Relationship between strike shift angle, boundary angle, and mining depth/loose layer thickness.

As can be seen in Fig 7, the strike shift angle and strike boundary angle increased when the ratio of mining depth minus the working face’s breadth to the loose layer’s thickness was increased.

#### Principle of GA-ELM neural network model

Due to the complex and diverse influencing factors of coal pillar retention parameters, the data-driven neural network model does not require an in-depth understanding of coal pillar retention parameters and can establish correlation patterns of relevant feature variables for similar geological formations through supervised learning of data samples. This approach exhibits good applicability and application prospects [39–43].

**Fig 8:**
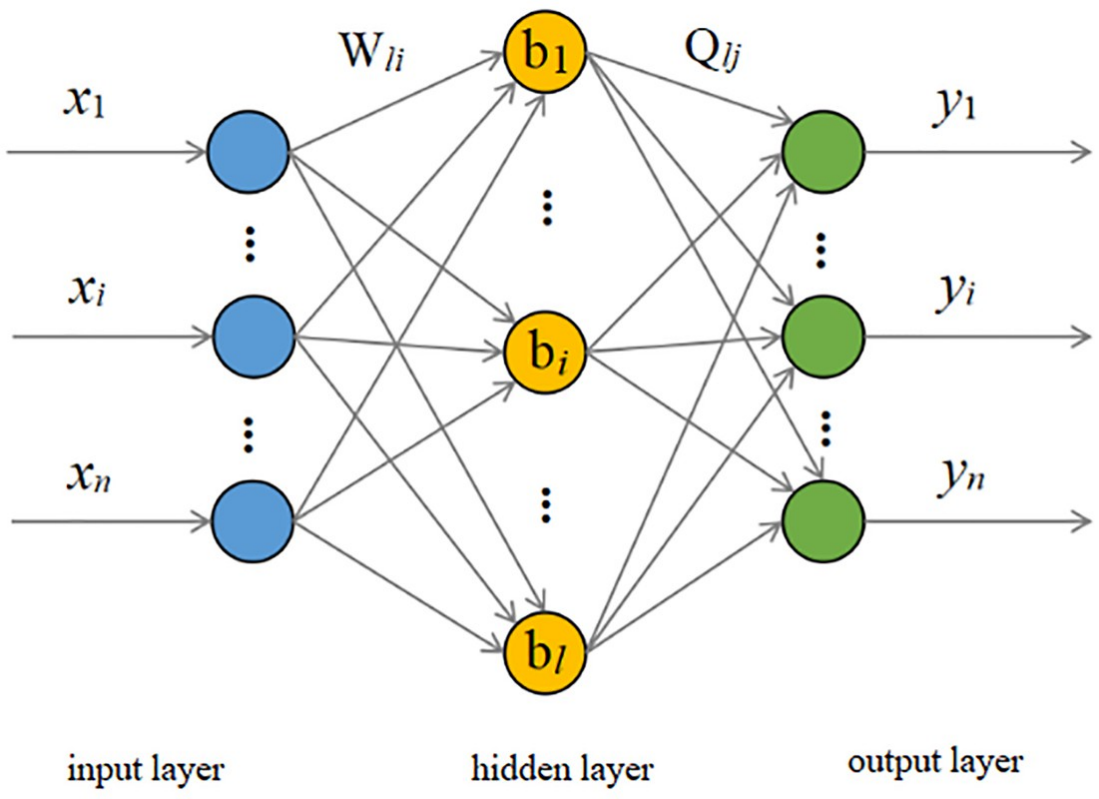
ELM neural network topology.

*ELM fundamentals*. The ELM neural network comprises a feedforward neural network with a single hidden layer. In contrast to the conventional iterative neural network, the ELM network is capable of addressing regression prediction problems with greatly reduced execution time by randomly selecting input layer weights, thereby improving the learning efficiency of the algorithm while offering the benefits of a simplified structure, high learning efficiency, and superior generalization capabilities. The topology of ELM is shown in Fig 8.

In Fig 8, *W*_*l*i_ and Q_*lj*_ represent the connection weights between neurons:

$$W=\left[\begin{array}{cccc} W_{11} & W_{12} & \cdots & W_{1i}\\ W_{21} & W_{22} & \cdots & W_{2i}\\ \vdots & \vdots & \cdots & \vdots \\ W_{l1} & W_{21} & \cdots & W_{li} \end{array}\right],$$ (14)

$$Q=\left[\begin{array}{cccc} Q_{11} & Q_{12} & \cdots & Q_{1j}\\ Q_{21} & Q_{22} & \cdots & Q_{2j}\\ \vdots & \vdots & \cdots & \vdots \\ Q_{l1} & Q_{21} & \cdots & Q_{lj} \end{array}\right].$$ (15)

Let the threshold b of the implicit layer neuron be $\text{b}=\left[\begin{array}{cccc} b_{1} & b_{2} & \cdots & b_{l} \end{array}\right]$.

Let the training input sample be X and the output sample be Y. Then $\text{X}=\left[\begin{array}{cccc} x_{11} & x_{12} & \cdots & x_{1R}\\ x_{21} & x_{22} & \cdots & x_{2R}\\ \vdots & \vdots & \cdots & \vdots \\ x_{i1} & x_{21} & \cdots & x_{iR} \end{array}\right]$$\text{Y}=\left[\begin{array}{cccc} y_{11} & y_{12} & \cdots & y_{1R}\\ y_{21} & y_{22} & \cdots & y_{2R}\\ \vdots & \vdots & \cdots & \vdots \\ y_{j1} & y_{21} & \cdots & y_{jR} \end{array}\right]$.

R is the number of samples.

Let the activation function of the neurons in the hidden layer be g(*x*). Then, the network output T is

$$\text{T}={\left[\begin{array}{cccc} t_{1} & t_{2} & \cdots & t_{R} \end{array}\right]}_{j\times R}t_{m}=\left[\begin{array}{c} t_{1m}\\ t_{1m}\\ \vdots \\ t_{nm} \end{array}\right]=\left[\begin{array}{c} \sum_{n=1}^{m}{\text{Q}}_{n1}\text{g}\left({\text{W}}_{n}x_{m}+{\text{b}}_{n}\right)\\ \sum_{n=1}^{m}{\text{Q}}_{n2}\text{g}\left({\text{W}}_{n}x_{m}+{\text{b}}_{n}\right)\\ \vdots \\ \sum_{n=1}^{m}{\text{Q}}_{nm}\text{g}\left({\text{W}}_{n}x_{m}+{\text{b}}_{n}\right) \end{array}\right],\text{M}=1,2,\ldots ,\text{R}.$$ (16)

Eq (16) can be transformed into HQ = T

Where

$$\text{H}=\left[\begin{array}{cccc} \text{g}\left({\text{W}}_{1}x_{1}+{\text{b}}_{1}\right) & \text{g}\left({\text{W}}_{2}x_{1}+{\text{b}}_{2}\right) & \cdots & \text{g}\left({\text{W}}_{l}x_{1}+{\text{b}}_{l}\right)\\ \text{g}\left({\text{W}}_{1}x_{2}+{\text{b}}_{1}\right) & \text{g}\left({\text{W}}_{2}x_{2}+{\text{b}}_{2}\right) & \cdots & \text{g}\left({\text{W}}_{l}x_{2}+{\text{b}}_{l}\right)\\ \vdots & \vdots & \cdots & \vdots \\ \text{g}\left({\text{W}}_{1}x_{\text{R}}+{\text{b}}_{1}\right) & \text{g}\left({\text{W}}_{2}x_{\text{R}}+{\text{b}}_{2}\right) & \cdots & \text{g}\left({\text{W}}_{l}x_{\text{R}}+{\text{b}}_{l}\right) \end{array}\right].$$ (17)

After the weights and thresholds are determined, the output matrix can be uniquely determined, and the connection weights *β* between the input and output layers can be obtained using the following equation:

$$\tilde{\beta }=\underset{\beta }{\text{min}}\|\text{HQ-T}\|,$$ (18)

Where ∥•∥ is the norm, and the final solution is

$$\hat{\text{Q}}={\text{H}}^{*}\text{T}$$ (19)

**Fig 9:**
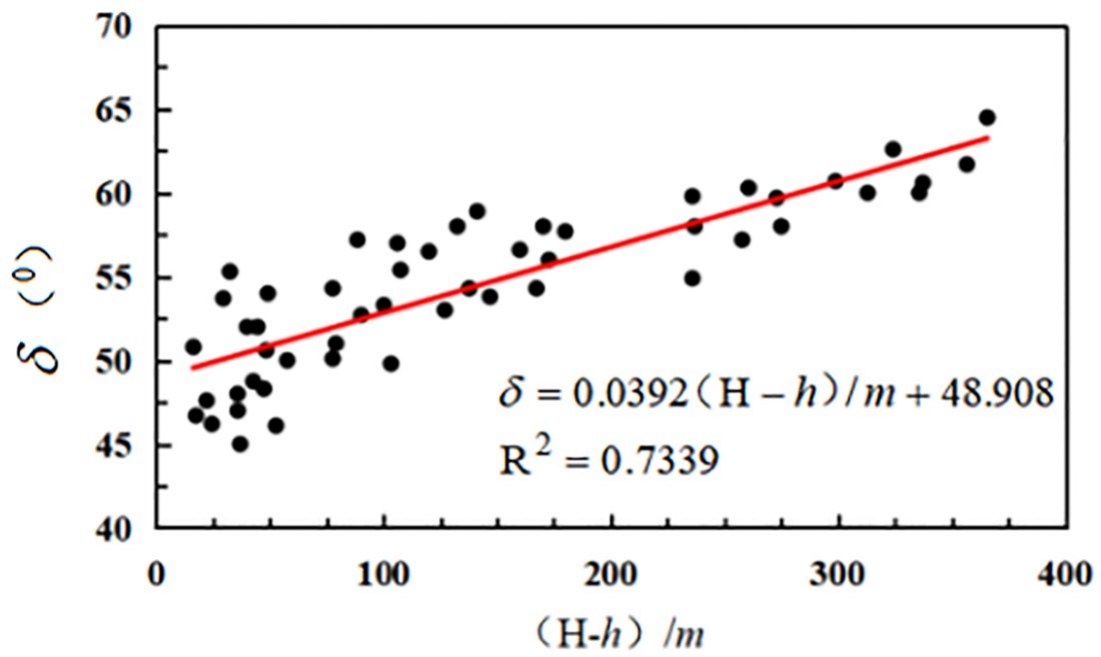
GA algorithm.

*Principle of genetic algorithm*. GA is an adaptive search methodology that emulates the biological process of genetic variation observed in natural populations. By following the principle of “survival of the fittest,” it facilitates optimal solution search and transforms the given problem into a natural selection and evolution process with robust global search capabilities. This process is illustrated in Fig 9.

#### Algorithm construction of GA-ELM model

**Fig 10:**
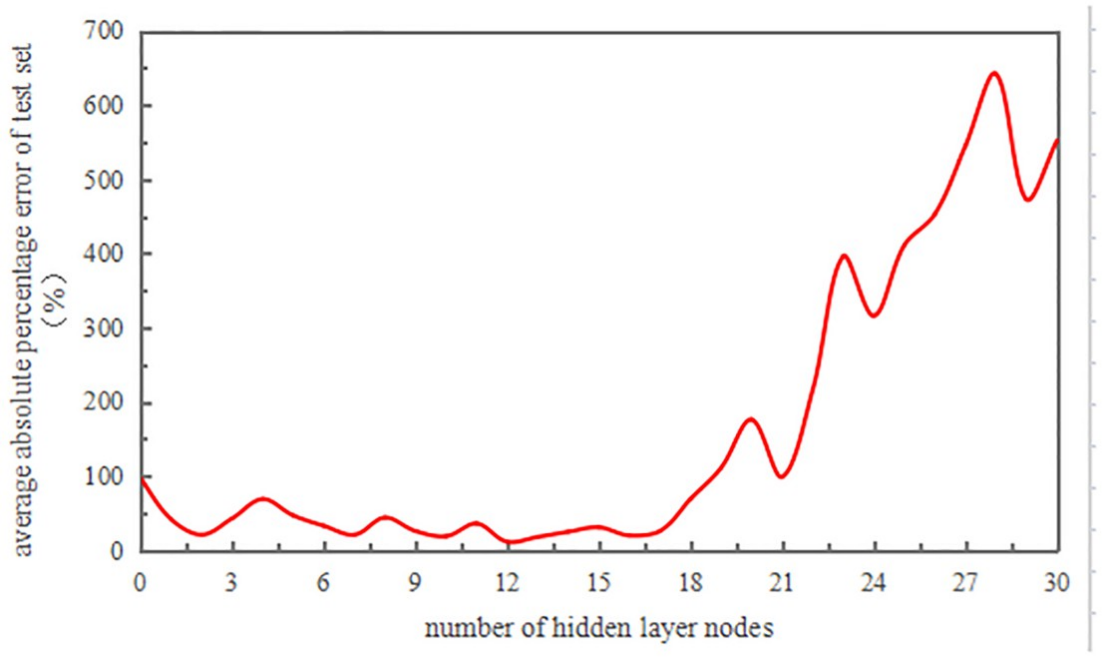
GA-ELM algorithm.

Although the ELM neural network can accurately predict most parameters, the problem of overfitting is frequently encountered in real-world applications, resulting in the poor fitting ability and stability of the algorithm model. The GA’s optimization-seeking function can be utilized to obtain optimal weights and thresholds for the ELM neural network. This approach effectively addresses the problem of poor generalization performance exhibited by the ELM neural network. This process includes the following steps (Fig 10):

Step 1: Input sample data.
Step 2: Encode the randomly generated weights and thresholds of the ELM neural network to serve as the initial population for the GA.
Step 3: Calculate the fitness value of the population.
Step 4: Update and evolve the population.
Step 5: After iterations, obtain the most appropriate weights and thresholds.
Step 6: Assign the optimal weights and thresholds to the ELM model for prediction output.

## Results

### Estimation of the combined prediction model

#### Experimental data

**Fig 11:**
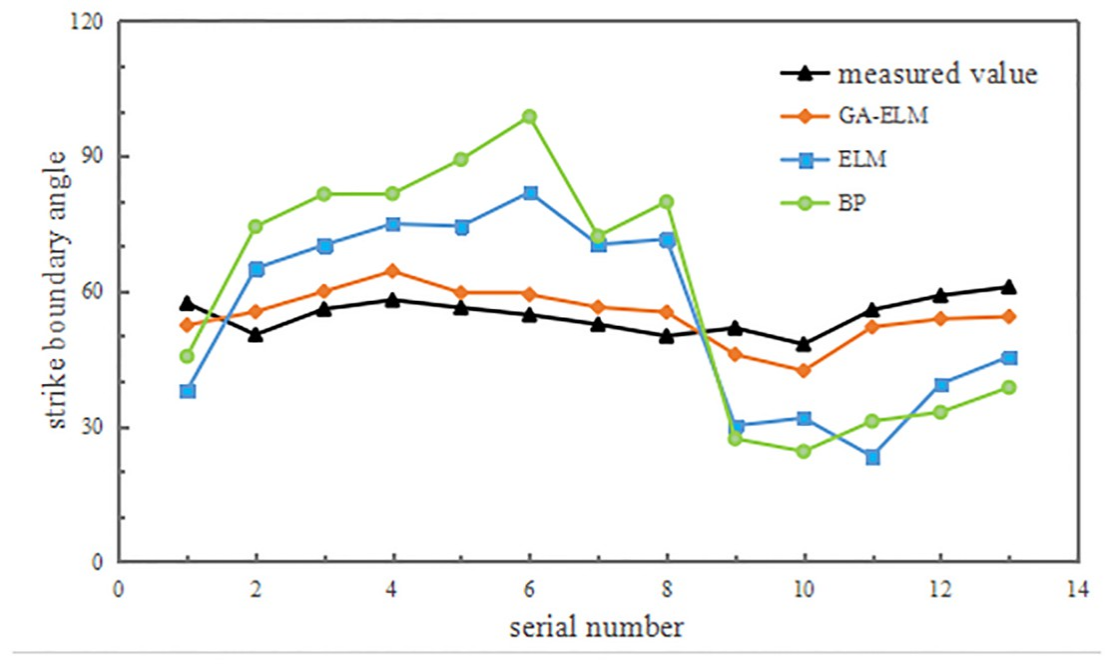
Regression model.

To evaluate the generalization performance of the GA-ELM model, we used the strike boundary angle as an example for analysis. We collected 65 sets of observation station data. As discussed earlier, the coal mining depth, loose layer thickness, coal seam thickness, and other factors affect the retention characteristics of coal pillars. We established a mathematical regression model by using the strike boundary angle as an example. The results are displayed in Fig 11 [44, 45].

#### GA-ELM model prediction

The choice of activation function affects the ELM model’s generalization performance. Commonly used activation functions include the sigmoid function, sin function, and hardlim function. Among them, the sigmoid function is preferred because of its monotonically increasing nature, which aligns with the prediction curve of the regression model. The accuracy of prediction results can be influenced by the number of input layers. Therefore, while determining the number of input and output layers and other parameters, we consider different numbers of input layers. We evaluated the accuracy of the results by using the mean absolute percentage error (MAPE) of the prediction results, as illustrated in Fig 10.

The accuracy achieved by different numbers of input layers varied greatly (Fig 10). When the number of input layers was set as 12, the accuracy was the highest (11.59%). As a result, the number of input layers was set as 12 in this paper, and the other parameters were set as stated in Table 6.

**Table 6:** GA-ELM parameter settings.

Parameter	Numerical value
**GA population size**	80
**Iteration Times**	100
**Crossover probabilities**	0.6
**Variation probability**	0.01
**Number of ELM input layers**	3
**Number of ELM implicit layers**	12
**Number of ELM output layers**	1
**Activation functions**	Sigmoid

In total, 65 sets of data were used to obtain predictions based on the set parameters, with 80% of the data used for modelling and the remaining 20% for validation. The results are presented in Fig 11.

As can be seen in Fig 11, the prediction values obtained using the GA-ELM model exhibited closer proximity to the measured values, whereas the ELM model and BP model yielded very few results with high accuracy, with larger variances and poorer alignment with the measured values observed in most cases. To assess the prediction performance of the GA-ELM, ELM, and BP models, we calculated accuracy evaluation indices for prediction values obtained using these models. We used two indices for comparison, namely the mean absolute error (MAE) and the MAPE: $\text{MAE}=\frac{1}{n}\sum_{i=1}^{n}\left|{x^{\prime }}_{i}-x_{i}\right|$, $\text{MAPE}=\frac{1}{n}\sum_{i=1}^{n}\left|\frac{{x^{\prime }}_{i}-x_{i}}{x_{i}}\right|\times 100\%$, where ${x^{\prime }}_{i}$ is the predicted value, *x*_*i*_ is the measured value, and *n* is the number of predicted samples.

The MAE index represents the average error between the predicted and actual values, with a smaller value indicating higher prediction model stability. In contrast, the MAPE index reflects the average deviation rate between the predicted and true values of the model. A smaller MAPE value indicates a closer match between the predicted and true values, indicating better model fitting. The calculated results are presented in Table 7.

**Table 7:** Accuracy of prediction results.

Prediction model	GA-ELM	ELM	BP
**MAE**	4.97	19.65	25.58
**MAPE**	9.13%	36.07%	47.07%

As can be seen in Table 7, the accuracy index of the prediction results was GA-ELM > ELM > BP, and the GA-ELM prediction model exhibited the highest overall accuracy despite some fluctuations in individual prediction results. The model’s overall prediction results were consistent with the actual measured values of the coal pillar retention parameters, demonstrating the superiority and accuracy of the proposed GA-ELM prediction model.

## Discussion

### Conclusion

Through an in-depth analysis of actual measurement data from the Huainan coal mine, we have successfully determined coal pillar retention parameters. Our study demonstrates scientific novelty in several key aspects:

Firstly, our method integrates the definition of coal pillar retention, requirements from the "Three Regulations," and geological mining conditions. This holistic approach enhances the precision of coal pillar retention parameter determination, providing valuable insights for the scientific and practical advancement of coal mining engineering design.

Secondly, the introduction of the MATLAB software platform and TLS algorithm for program validation showcases the stability and accuracy of our methodology through example analysis. This not only broadens the methodological scope but also enhances the reliability of results, offering an innovative technical path for solving similar problems in the future.

Additionally, our use of mathematical regression analysis to establish a linear fitting model quantitatively links coal pillar retention parameters to geological mining conditions. This step enriches our scientific understanding of coal pillar retention and provides robust support for more accurate predictions and adjustments.

Despite the significant scientific novelty achieved in our study, it is essential to acknowledge certain limitations. Firstly, our data is sourced exclusively from the Huainan coal mine, potentially restricting the generalizability of our findings to other regions. Secondly, while our proposed model excels in predicting coal pillar retention parameters, real-world applications may necessitate consideration of additional influencing factors.

In conclusion, by processing data from diverse scenarios, this paper offers technical support and lays the groundwork for more precise determination of coal pillar parameters. Our research holds unique value in terms of scientific and practical contributions, providing a fresh perspective for coal mining engineering design and production practices. Future studies could expand the geographical scope and further refine the model’s applicability and robustness.
